# Methylation Potential of Mercury Impacted Pipeline Scale

**DOI:** 10.3390/toxics14070612

**Published:** 2026-07-13

**Authors:** Hasti Ziaei Jam, Paul Bireta, Danny Reible

**Affiliations:** 1 Department of Civil, Environmental and Construction Engineering, Texas Tech University, Lubbock, TX 79409, USA; hasti.ziaee@gmail.com; 2 Chevron Corporation, 1500 Louisiana Street, Houston, TX 77004, USA; pbireta@chevron.com

**Keywords:** mercury, methylmercury, oil and gas, pipeline scale

## Abstract

This study assessed the leachability and methylation potential of total mercury (THg) associated with internal deposits in subsea oil and gas pipeline segments that are abandoned on the seafloor. The studies were conducted for up to 28 weeks under anaerobic conditions in static microcosms in which mercury-contaminated scale was exposed directly to sediments. The scale had an XRF-measured surficial THg concentration ranging between 15 and 3000 µg/cm^2^. Release to sediment contacting the scale was rapid with no significant changes noted in THg concentrations after 6 weeks. THg released to sediments from intact pipeline coupons with low THg (15.5–124 µg/cm^2^) released an average of 16% of the THg mass present in the coupon into the sediments while those with higher THg (457–1367 µg/cm^2^) released an average of just 0.85% of the THg present in the coupon. Additional studies were conducted with all scale shaved into microcosms to simulate rapid and complete corrosion of the pipeline. The resulting sediment concentrations showed that the XRF-measured surficial THg accounted for all of the THg in the scale. In both sets of experiments, peak methyl mercury (MeHg) sediment concentration (measured at 8 and 12 weeks) averaged just 0.05% of the THg in the sediment. MeHg in the sediment was not correlated with the mass of THg measured in the scale and showed only a weak correlation with sediment THg suggesting that much of the THg is unavailable for release and methylation. The sediment MeHg concentration was positively correlated with leachable THg measured as filtered THg (passing 0.45 µm filter) (R^2^ = 0.81). The methods presented here provide a means of assessing mercury release from pipeline scale and its implications in other environments.

## 1. Introduction

Fossil fuels, such as crude oil, natural gas, and coal, can contain trace levels of naturally occurring mercury (Hg) [1,2]. There are different theories about the origin of this mercury, with some proposing that it accumulates in organisms that contribute to the formation of natural gas and hydrocarbons [3], while others suggest that it enters these reservoirs from source rocks, mercury-rich fluids, and mantle-derived elemental mercury (Hg^0^) that is subsequently scavenged by petroleum [4,5]. Mercury is a common contaminant of subsurface fluids, predominantly as Hg^0^ and mercury sulfide (HgS) [6]. This mercury can be brought to the surface, for example, during oil and gas extraction, where it can accumulate through various processes, including deposition of Hg^0^, adsorption onto steel surfaces, and deposition within inorganic precipitates as pipe scale [7]. Mercury concentrations in gas, condensate, and crude oil exhibit significant variation across different regions and time periods. For instance, Kho et al. (2022) [7] reported that mercury levels in natural gas produced in China can range from 0.01 µg/cm^3^ to 1000 µg/cm^3^ and mercury concentrations in crude oil produced in Thailand can vary from <0.01 µg/kg to 1000 µg/kg. Ryzhov et al. showed that mercury concentrations in natural gas can fluctuate over time, ranging from 1.6 µg/cm^3^ to approximately 25 µg/cm^3^ over a span of 20 years at a single location [8].

The presence of hydrogen sulfide (H_2_S) in water creates a weak acidic environment that releases hydrogen ions, making the fluid containing oil and water corrosive, with iron sulfides (FeS_x_) forming as the primary corrosion products. Iron sulfide (FeS) can bind with mercury ions (Hg^2+^) present in oil and co-precipitate as HgS [7,9]—a compound frequently identified as the predominant form of mercury within corrosion layers [10,11,12]. Microscopic analysis of pipeline cross-sections has confirmed that HgS deposits as a distinct phase on the immediate gas-contacting surface, layered over an underlying iron oxide scale [13]. Interestingly, even on corrosion-free carbon steel surfaces, long-term mercury accumulation can reach levels comparable to those found on corroded steel; the critical difference lies in the uptake kinetics—accumulation on corroded surfaces occurs rapidly within days, whereas corrosion-free surfaces accumulate mercury more gradually over several months [11]. However, evidence suggests that mercury only deposits on the surface of pipelines, and does not penetrate into uncorroded steel [14,15]. This is mechanistically supported by Wilhelm [13], who noted that the large atomic size of mercury prevents it from diffusing into the interstitial spaces of the steel lattice. Consequently, while operational mitigation strategies like internal coatings and corrosion inhibitors are highly effective at preventing active corrosion [16,17], they may ultimately alter the kinetics of mercury uptake rather than eliminate it entirely. To manage these risks throughout the operational lifecycle, the industry actively utilizes models to predict mercury scale accumulation and mitigate future decommissioning liabilities [9].

Decommissioning of oil and gas production facilities typically includes removal and reuse or recycling of process structures. Depending on site specific conditions, decommissioning strategies sometimes includes leaving a portion of disconnected steel pipelines on the seafloor to allow for natural decay [18,19]. In fact, recent research indicates that such in situ decommissioning can provide distinct environmental benefits, including the formation of artificial reefs that enhance local marine biodiversity and serve as productive fishery locations [20]. However, a contrasting potential concern for trace levels of mercury that may be present in corrosion layers, is bacterial transformation to organic mercury species, which can bioaccumulate and biomagnify in fish tissue and ultimately pose significant health risks to humans [21,22,23]. To evaluate the methylation potential of mercury-contaminated sediments, microcosm experiments have long served as a standard methodology [24,25,26]. Our previous work demonstrated that mercury bioavailability is highly dependent on chemical speciation; in sediment microcosm experiments, matrixes dominated by metacinnabar (β-HgS) were found to be mostly inert, with <0.002% available for methylation, whereas sediment with predominant mercury-thiol (Hg (SR)_2_) complexes reached up to 0.1% methylation [27]. Speciation of Hg and its relationship to methylation remains an important area of research. Microcosms provide a tool to assess methylation without knowing or understanding the complex relationship between Hg species and methylation. Moreover, because subsea pipeline scale is chemically and structurally distinct from natural sediments, its methylation potential cannot be accurately predicted from sediment baselines alone [11,20,28]. We chose instead to use an experimental assay in microcosms to evaluate mercury availability and methylation potential from Hg contaminated scale.

This study evaluates the methylation potential for mercury present in pipeline corrosion material in simulated marine environments using coupons cut from mercury impacted oil and gas pipelines. In particular, the study seeks to evaluate whether a significant fraction of mercury in the scale is unavailable for release into the sediments and methylation. A simple laboratory assay was developed and applied to evaluate a variety of pipeline coupons under representative conditions that were designed to provide a conservative indication of mercury release and methylation potential from the pipeline scale. The results indicate the mercury availability and methylation potential from a particular sediment, but it is hoped the methodology can be applied in similar situations.

## 2. Materials and Methods

### 2.1. Materials and Analytical Methods

The sediment employed in this study consisted of a fine-grained, unimpacted, saturated marine sediment with background Hg concentrations of approximately 0.025 (±0.005) mg/kg. The sediment contained 1.8% organic carbon and was dominated by fine silt (50%, 2–45 µm), followed by coarse silt and sand (25%, >45 µm), and clay (25%, <2 µm). This sediment was collected from the same oil and gas producing area as the contaminated pipelines tested but sufficiently distant to be unimpacted. Filtered porewater (0.45 µm) contained 4.56 (±4.81) ng/L Hg, leading to a sediment–water partition coefficient for THg of 7550 (±3170) L/kg, similar to the partition coefficient measured by [29,30].

Pipeline samples consisted of approximately 2.35 cm × 2.35 cm and 1.5 cm thick steel coupons that were cut out of subsea decommissioned oil pipelines. The surface concentrations of THg in the pipeline scale was estimated by X-ray fluorescence (XRF) in surface mode (conducted separately by project sponsor Chevron), indicating values ranging between 15 and 3000 µg/cm^2^. The mass of Hg on the surface of the coupon was calculated by multiplying the XRF value by the area of the pipeline interior surface. The coupons were either placed directly into 60 mL jars containing 30 g of marine sediment with ~40% moisture content, or the scale was shaved from the coupons and added to the jars. The microcosms were maintained in an anaerobic chamber and selected microcosms were sacrificed at regular intervals and THg and MeHg formation were measured. These experiments were conducted with in an anaerobic chamber to simulate biogeochemical conditions representative of buried sediment and to maximize the potential for methylation (which occurs under reduced conditions). The choice of 30 g of wet sediment (~1 g/cm^3^) was based on the concept that the pipeline coupons were approximately 6 cm^2^ of exposed area and that bioturbation would tend to keep any Hg released well-mixed over a biologically active zone of at least 5 cm. Deeper or lateral mixing which would dilute the mercury concentrations was not considered to provide a conservative indicator of mercury impacts on the sediment. The scale of the experiment was chosen to allow easy replication.

The sediment samples were digested and analyzed for total Hg using EPA Method 1631 [31], and for MeHg using EPA Method 1630 [32], Brooks Rand Merx-T cold vapor atomic fluorescence spectroscopy (CVAFS) system (Brooks Rand Instruments, Seattle, WA, USA) was used for THg, whereas the MeHg samples were analyzed by Brooks Rand Merx-M CVAFS system (Brooks Rand Instruments, Seattle, WA, USA).

For THg the instrument detection limit was 2.5 ng/L Hg. Blanks were all below detection while the SRM recovery was 91.5 ± 9.5% and the recovery for the 10 μg/L total Hg standards was 104 ± 4%. The detection limit for sediment total Hg using this method was determined to be 0.33 μg/kg (dry weight).

For MeHg the instrument detection limit was 1 ng/L MeHg. Method and reagent blanks were all below detection while the recoveries for the European Reference Material CC580 was 72% ± 16% and for the 10 μg/L MeHg standard 96% ± 10%. The detection limit for sediment MeHg using this method was calculated to be 0.056 μg/kg (dry weight).

### 2.2. Microcosms to Assess Mercury Release and Methylation

The purpose of this experiment was to assess the Hg release and methylation from the scale from pipeline coupons into sediment with which it was in direct contact.

An initial experiment was conducted comparing Hg release from the coupons with and without the cut edges covered by epoxy to check whether the cut surfaces artificially leached extra mercury. Ten coupons were selected to evaluate the effect of edges on Hg release at 4, 8 and 10 weeks. Four coupons were coated on the sides, excluding the main surface, using epoxy (JB Weld Steel Reinforced Epoxy). Each epoxied coupon was paired with another non-epoxied coupon with similar Hg loading as measured by XRF. Two pairs of coupons had XRF-measured loading of 145–150 µg/cm^2^ and two epoxied and four non-epoxied coupons had XRF-measured loading of 46–61 µg/cm^2^. Microcosms were kept in an anaerobic chamber and three control sediment samples without coupons were prepared for each timepoint for reference. At each time point, the coupons were removed, sediments homogenized, and five replicate subsamples collected for analysis. Since the comparison of epoxied and non-epoxied coupons showed no meaningful difference in Hg release between epoxied and non-epoxied coupons under anaerobic conditions, all subsequent experiments were conducted using non-epoxied coupons only. The subsequent experiments used 36 coupons with varying Hg scale concentrations as measured by XRF.

The 36 coupons were in two broad groups, 9 samples exhibiting high THg scale (ranging from 457 to 1367 µg/cm^2^) and 27 coupons with low Hg scale concentration (ranging from 15.5 to 124 µg/cm^2^). The coupons were placed in jars containing 30 g (wet weight) of marine sediment at in situ density and moisture content as noted above. Both the jars and the subsequent incubation were conducted in an anaerobic chamber to maximize methylation.

The interior surface of the coupons was positioned facing the sediment to allow direct contact of the contaminated scale and sediment and simulate a cut or partially decomposed pipeline. Preliminary studies suggested that MeHg concentration peaked after approximately 8–12 weeks of incubation, and samples were analyzed for MeHg at those timepoints. However, the low Hg level samples were run for a longer period to identify any potential increase in THg release over time. The low Hg level scenario contained 9 sets of coupon/sediment samples in triplicate collected at 2, 4, 6, 8, 12, 16, 20, 24, and 28 weeks. The high Hg level scenario contained 3 sets of coupon/sediment samples in triplicate collected at 2, 8, and 12 weeks. No significant changes in THg sediment or filtered porewater concentrations were noted after 6 weeks. Control jars without coupons were also prepared for the 9 sampling time points.

At each timepoint, after removing the coupons from the jars, sediments were thoroughly mixed and homogenized. Subsequently five replicate samples were collected from each individual jar, and sediment porewaters were extracted by centrifugation of the remaining sediment at 5000 rpm for 30 min and at 4 °C in a Beckman Coulter centrifuge, followed by filtration using 0.45 µm PES filters. The collected samples were analyzed for THg in the solid phase and filtered water phase, MeHg in the sediments was measured at week 8 and 12.

### 2.3. Shaved Scale Leaching Batch Experiment

This experiment was to represent the leaching of Hg from a fully corroded and collapsed pipeline where all of the scale is incorporated into the sediments. Although this process may take decades or longer, it was essentially instantaneous in the experiment. The corroded scale was shaved from 29 pipeline coupons using a carbon rasp file, weighed, and added into the jars containing sediment. Due to the increased mass of THg shaved into the sediments, the sediment mass was increased to 80 g wet weight. The XRF values in the scale on the coupons ranged from 55 to 3000 µg/cm^2^. The jars were initially fully mixed by a shaker and 5 replicates were subsampled from each jar (with each subsample approximately 0.5 g) for THg concentration analysis. The same jars were kept under anaerobic conditions for periods of 8 and 12 weeks to evaluate potential leaching and methylation of Hg from the shaved scales to the sediment. After 8 weeks the jars were opened in an anaerobic chamber, and before homogenization and sediment disturbance, redox potential, H_2_S, pH, and dissolved oxygen were measured using microelectrodes (purchased from Unisense, Aarhus, Denmark). Subsequently, sediment samples were thoroughly mixed within the anaerobic chamber and approximately 35 g of sediment from each jar was transferred into a 50 mL centrifuge tube. Sediment porewaters were extracted as described above. The same process was conducted at 12 weeks. THg in sediment and filtered porewater samples as well as MeHg concentration in solid samples were measured using the same methods as described above.

## 3. Results

### 3.1. Evaluation of Mercury Release from Cut Surfaces

To verify whether the cut surfaces artificially release extra mercury from the coupons into the sediment, leaching from the exposed cut edges was evaluated by comparing the leachability of coupons with epoxy-coated edges against those left uncoated. Accordingly, Table 1 shows the THg concentration in the sediment leached from both coated and uncoated coupons. To ensure a reliable comparison, samples were categorized based on similar baseline mercury loadings as measured by XRF values and analyzed at 4, 8, and 10 weeks. The results show some variability but demonstrate comparable levels of Hg in the sediment between the non-epoxied and epoxied samples timepoint. In particular, there was no evidence that the Hg release from the pipeline coupons was different between epoxied edges and those with exposed edges. We therefore expect that pipeline coupons without epoxied edges can be used as representative THg sources in studies of Hg release and methylation potential.

### 3.2. THg Released and MeHg Formed from Exposure of Coupon to Sediments

**Figure 1:**
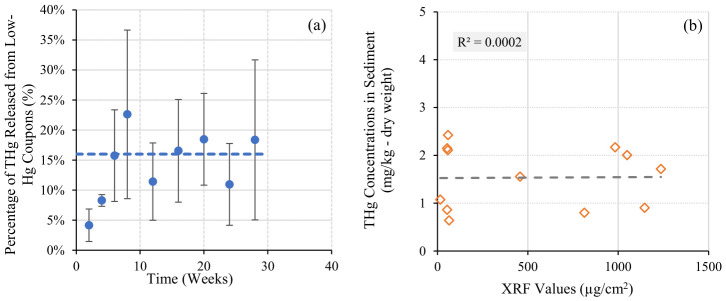
(**a**) Percentage of THg released from the low THg coupons over 28 weeks relative to the initial total mercury on the coupon, and (**b**) the THg sediment concentration resulting from exposure to coupons of different mercury content after 8 and 12 weeks. The blue dashed line in (**a**) indicates the mean percentage released across all time points (16%), while the grey dashed line in (**b**) represents the linear trendline (R^2^ = 0.0002). Error bars in (**a**) represent ± standard deviation.

The purpose of this experiment was to assess the Hg release and resulting methylation when the scale in pipeline coupons was simply exposed to sediments. Figure 1a shows the THg released into sediment and porewater from the 27 low concentration coupons maintained under anaerobic conditions over the 28 week study. Because the coupons varied in concentration, the average coupon concentration varied from sample to sample and the results are therefore normalized by the mass of THg in the scale in each coupon. In Figure 1a, the mass of THg in the sediment (concentration times sediment dry weight) is shown as a percentage of the mass in the coupon as estimated from XRF measurements. The figure shows that the mercury release from the coupons was rapid, achieving steady conditions after approximately 6 weeks in the microcosms, and that the final concentrations achieved was a small fraction of that which was present in the scale. For coupons with lower Hg content in the scale, an average of 16% of mercury in the scale was released to the sediments while for coupons with higher scale concentration, this percentage was reduced to 0.85%.

The observed THg concentration in the sediments for low (15.5–124 µg/cm^2^) and high (457–1367 µg/cm^2^) samples in 8 weeks led to sediment concentrations of THg of 1.46 ± 0.74 mg/kg and 1.49 ± 0.65 mg/kg, respectively. After 12 weeks these averages were essentially identical at 1.63 ± 0.77 mg/kg and 1.56 ± 0.73 mg/kg. Figure 1b shows, however, that the concentration observed in the sediment as a result of release from individual coupons was essentially independent of the mercury loading in those coupons as measured by XRF.

The sediment exposed to the contaminated scale thus developed THg concentrations in excess of 1 mg/kg. This can be compared to US NOAA screening levels for mercury in sediments of Effects Range Low (ERL) of 0.15 mg/kg and Effects Range Median (ERM) of 0.71 mg/kg (Sediment Quick Reference Table, SQuiRTs, https://repository.library.noaa.gov/view/noaa/9327, accessed on 28 June 2026). It should be emphasized, however, that this concentration was observed in a finite mass of sediment chosen to represent vertical mixing over an assumed 5 cm biological active zone without any lateral mixing. Any lateral mixing away from the contaminated pipeline would likely significantly reduce the resulting sediment concentration.

**Figure 2:**
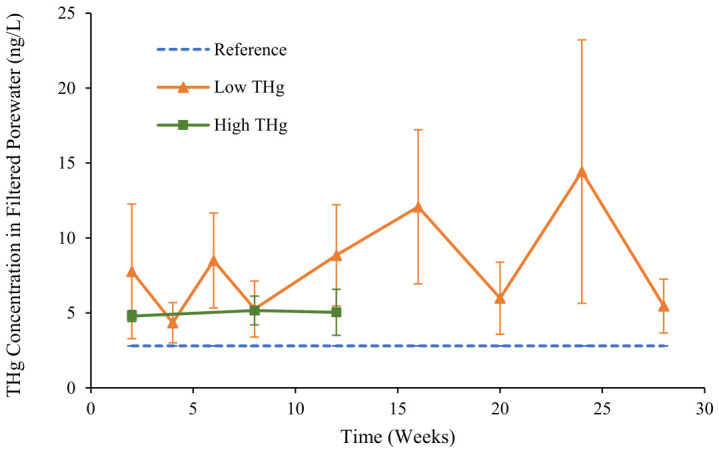
Measured THg concentration (±standard deviation) in filtered porewater for low and high THg coupon microcosms at each sampling period and average of reference sediments (2.8 ± 0.32 ng/L).

Average sediment porewater THg was also measured for all samples (Figure 2). The average THg in the porewater was 8 ± 3 ng/L for the coupons with lower XRF values, and 5 ± 0.2 ng/L for high XRF coupons. The reference sediment with only 0.03 mg/kg THg also had a porewater concentration of 4.6 ng/L. Thus, the mercury-contaminated scale resulted in minimal to modest differences in porewater mercury concentrations from uncontaminated sediment despite exceeding the ERL and ERM screening levels.

Based on preliminary measurements, the peak MeHg concentration occurred in these microcosms within 8 to 12 weeks after placing the coupons within the sediments. By that time, the THg release from the scale had occurred and the balance between methylation and demethylation achieved a quasi-steady MeHg concentration. For the samples with lower XRF values, the average MeHg concentration was 0.87 ± 0.63 µg/kg and 0.78 ± 0.38 µg/kg at 8 and 12 weeks respectively. For the samples with higher XRF values, the average MeHg concentration in sediment was 0.30 ± 0.24 µg/kg at 8 weeks and 0.72 ± 0.42 µg/kg at 12 weeks. The MeHg concentration measured after 12 weeks was approximately 0.11% of the THg concentration in the sediment, suggesting that much of the released Hg was not biologically available for methylating bacteria, even after release from the pipe scale. Previous studies suggest that much of the Hg in the pipe scale may be in the form of largely unavailable metacinnabar [7,10]. Together these results suggest that very little of the Hg in the coupons is released to the surrounding sediment and that this amount is not proportional to the total loading of Hg on the pipe scale.

MeHg concentrations in the contaminated pipeline coupons could also be compared to the reference sediment with an approximately 100 times lower THg concentration in sediment. As shown above, the porewater concentration in the reference sediment was only moderately lower than in the microcosms exposed to contaminated scale. In addition, the peak MeHg concentration in the reference sediments was 0.134 ± 0.07 µg/kg—only a factor of 2–6 times lower than the much more contaminated sediment (in terms of sediment THg concentration) exposed to the pipeline coupons. This further suggests that the THg in the contaminated scale was less available for release to water and to methylation than in other sediments with similar concentrations.

### 3.3. THg and MeHg in the Shaved Scale Experiment

**Figure 3:**
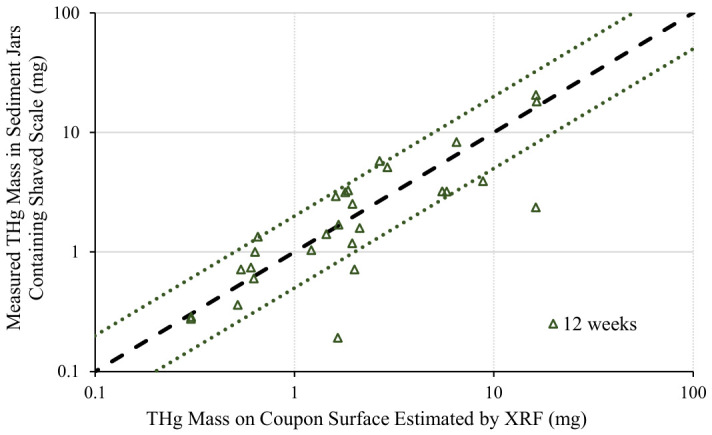
Comparison of measured THg mass in sediment jars after shaving of all scale into the jars and the predicted THg mass from XRF measurement after 12 weeks. The black dashed line represents the 1:1 ideal agreement line, while the green dotted lines indicate a factor of 2 difference from the expected THg based on XRF measurement.

In the final experiment, all of the pipeline scale on coupons was shaved into the sediment microcosm. This experiment was designed to simulate a conservative scenario where the pipeline had completely corroded, and all of the scale had been released into the sediment in a finely divided form. This is a process which may take decades to centuries to occur under field conditions but for the purpose of this experiment occurred instantaneously. The solid concentrations of THg were measured at the initial setup, 8 weeks, and 12 weeks. There was some variability in sediment Hg concentration in subsamples initially and at 8 weeks, but by 12 weeks of occasional mixing the Hg concentration was relatively uniform throughout the microcosms. The solid concentrations were multiplied by the total mass of sediment loaded to each jar to calculate the total mass of THg. The total mass of THg in coupon scale was estimated by multiplying the surface concentration measured by XRF and the surface area of the coupon (2.35 × 2.35 cm). Figure 3 shows the comparison between the measured THg mass versus the estimated THg mass with the solid line representing 1:1 and the two dotted lines showing the concentration ranging by a factor of 2. On average, the measured THg concentration in the sediments was 105% of that estimated by XRF at 12 weeks. This illustrated that the XRF measurement times coupon surface area was a good estimation of the total Hg in the pipeline scale.

**Figure 4:**
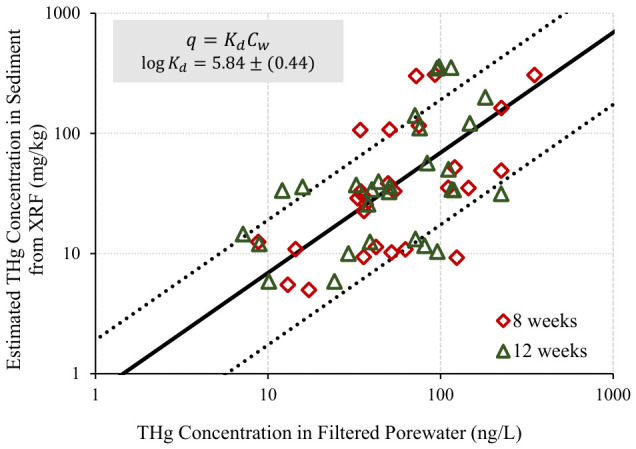
Comparison of the measured filtered porewater concentration versus estimated THg concentration from XRF measurement in 8 weeks (red diamonds) and 12 weeks (green triangles). The partition coefficient $K_{d}$ is fitted using the combined 8- and 12-week data. The solid black line represents the best-fit linear regression ($K_{d}=5.84$), while the dotted lines represent the standard deviation boundary ± 0.44.

Filtered porewater samples taken from each jar at 8 weeks and 12 weeks were measured and plotted against the XRF estimated total Hg mass in the scale (Figure 4). The average filtered porewater concentration was 76.8 (±77.2) ng/L at 8 weeks and 73.4 (±52.9) ng/L at 12 weeks with the variation associated with the different THg concentrations in the sediment. The sediment–water partition coefficients of THg for sediments containing shaved scale were found to range from 5.02 to 6.52 (log units of L/kg) with an average of 5.84 ± 0.44.

**Figure 5:**
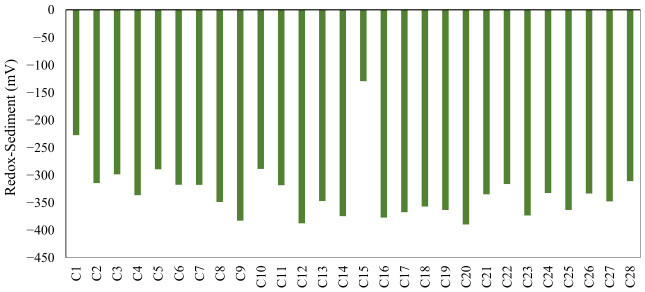
Redox conditions in all microcosms containing sediment and coupon shavings (12 weeks).

**Figure 6:**
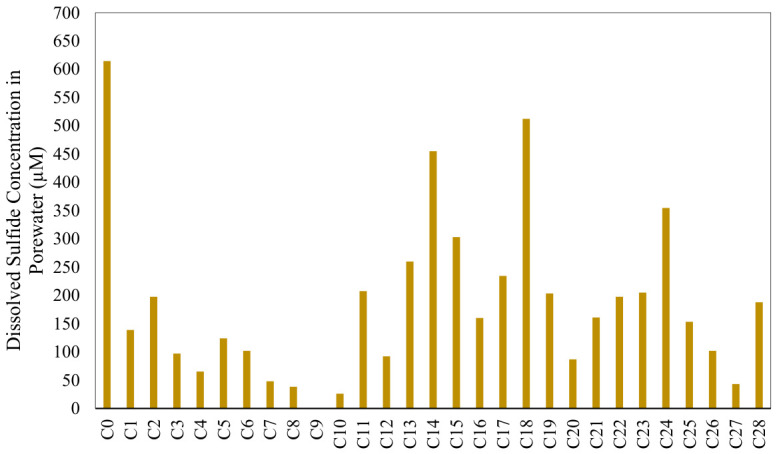
Dissolved Sulfide level in microcosms containing sediment/shaved-scale mixture.

After both 8 and 12 weeks, all samples showed evidence of reduction with all exhibiting redox conditions of <−100 mV (Figure 5). All samples also showed dissolved sulfide in porewater samples, typically above 50 µM after 8 weeks and above 100 µM after 12 weeks (Figure 6). MeHg concentrations after 8 weeks were similar to those after 12 weeks (average of 19 µg/kg vs. 22 µg/kg), indicating that extending the assay beyond 8 weeks provided little additional methylation. The MeHg/ THg ratio in the sediments after 12 weeks averaged approximately 0.05% with a maximum value of 0.21%, suggesting again that very little of the Hg in the sediment was methylated. Note that the MeHg/THg ratios in sediments were similar but slightly lower (0.01–0.08%) when the scale was fully shaved into the sediments.

**Figure 7:**
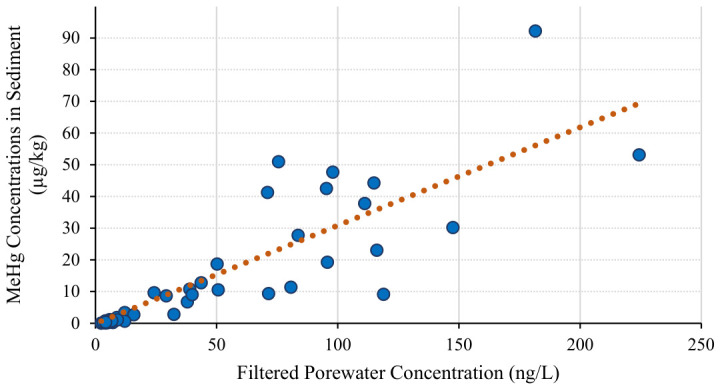
Correlation between the methylated fraction of THg in the sediment (%MeHg/THg) and the fraction of dissolved THg filtered by 0.45 µm filters over THg in sediment (FW Hg%). Dotted line is ${\left[MeHg\right]}_{sed}=0.31\;\left(\pm 0.023\right)\times {\left[THg\right]}_{FW}$, R^2^ = 0.81 (MeHg in μg/kg and THg in ng/L).

The filtered porewater THg concentrations were tested as a predictor of MeHg production and concentration. The correlation between sediment MeHg concentration and filtered THg—from both initial experiments with natural mercury release and experiments with shaved coupons—yielded the relationship ${\left[MeHg\right]}_{sed}=0.31\;\left(\pm 0.023\right)\times {\left[THg\right]}_{FW}$ with an R^2^ = 0.81 (Figure 7), where ${\left[MeHg\right]}_{sed}$ is the sediment concentration in μg/kg and ${\left[THg\right]}_{FW}$ is the porewater filtered concentration in ng/L. This suggests that filtered porewater concentrations are a potential indicator of Hg bioavailability whereas total sediment Hg was not. The filtered porewater concentrations provide an indication of the available Hg and excludes Hg that is particle associated or associated with deposited sediment. The slope of the correlation, 310 L/kg, represents a balance between formation of MeHg from available Hg and demethylation with associated formation and demethylation rate constants, $k_{f}$ and $k_{d}$.

$$R_{MeHg}=k_{f}\;{\left[THg\right]}_{FW}-\frac{k_{d}}{K_{MeHg-w}}{\left[MeHg\right]}_{sed}$$

Here it is assumed that MeHg is in local equilibrium with the sediment (with partition coefficient $K_{MeHg-w}$) and that the respective water concentrations control net mercury methylation and demethylation, respectively. At equilibrium,

$${\left[MeHg\right]}_{sed}=\frac{k_{f}K_{MeHg-w}}{k_{d}}{\left[THg\right]}_{FW}$$

The slope of the relationship therefore represents the combination of forward methylation rate times the MeHg sediment–water partition coefficient divided by the demethylation rate constant. The fact that Figure 7 shows an approximate linear relationship suggests that these factors are approximately constant in the sediments and conditions of the experiments. The small variations around the best fit line in Figure 7 might reflect variations in sediment or lack of reaction or sorption equilibrium in particular microcosms. The variations, however, are generally less than a factor of two in the entire set of experiments.

## 4. Conclusions

This study aimed to explore potential environmental impacts of mercury released during corrosion of decommissioned subsea pipeline segments that are abandoned in place on the seafloor. A batch microcosm experiment under static anaerobic conditions was conducted with a marine sediment at near in situ density and moisture content and using a volume of sediment similar to the expected volume to which a pipeline might be exposed. It provided the possibility to examine a wide range of Hg impacted coupons with varying concentrations (15–3000 µg/cm^2^). Results indicated that THg on the surface of the pipeline scale (measured by XRF) is not a good indicator of leachable Hg and Hg methylation potential. The experiments demonstrated that a small fraction of the THg in the scale would leach into the sediments under normal conditions (<1% for highly contaminated scale) and regardless of whether the THg in the scale is released by contact with a coupon or shaved into the sediments, the concentration of MeHg in the sediments was an average of 0.05% and a maximum of 0.21% of the THg. Methylation is better indicated by leachable Hg and especially dissolved Hg (as indicated by filtered porewater samples). The relationship between MeHg in these sediments and filtered porewater THg was found to be MeHg (Sediment µg/kg) = 0.31 (±0.023) THg (filtered, ng/L), R^2^ = 0.81. This may suggest that other sediments may also show correlations between MeHg and dissolved/filtered aqueous sample THg. The small amount of mercury released from the pipeline scale combined with low extent of methylation suggests that at least in this system, the risks of the mercury-contaminated scale in the pipeline are small even though the observed sediment concentrations might exceed common sediment screening benchmarks. The measured concentrations of THg and MeHg resulting from exposure to the contaminated pipeline scale also represent maximum values that would be reduced by mixing and sediment transport away from the source of contamination. The methods outlined in this manuscript also indicate how to assess such risks in other systems via a relatively simple approach designed to maximize conditions for mercury release into the sediments and encourage methylation.

## Figures and Tables

**Table 1:** Comparison of Hg leachability between epoxy-coated coupons (except the main surface) and the non-epoxied coupons (the standard deviation is calculated from five replicates).

ID	Type	XRF (µg/cm^2^)	Week Analyzed	Sediment THg Concentration (mg/kg-Dry)	Standard Deviation
EP1	Epoxied	148	4	1.12	0.12
NP1	Non-epoxied	145	4	0.78	0.09
					
EP2	Epoxied	46	8	2.62	0.70
EP3	Epoxied	56	8	3.70	0.29
NP2	Non-epoxied	53	8	2.48	0.47
NP3	Non-epoxied	53	8	4.21	1.00
NP4	Non-epoxied	57	8	1.77	0.36
NP5	Non-epoxied	61	8	3.65	1.61
					
EP4	Epoxied	150	10	1.14	0.15
NP6	Non-epoxied	150	10	1.40	0.18
					
Ref 1	Control		4	0.027	0.002
Ref 2	Control		8	0.027	0.005
Ref 3	Control		10	0.034	0.002

## Data Availability

The original contributions presented in this study are included in the article. Further inquiries can be directed to the corresponding author.

## Abbreviations

The following abbreviations are used in this manuscript:

β-HgS	metacinnabar
CVAFS	cold vapor atomic fluorescence spectroscopy
ERL	effects range low
ERM	effects range median
FeS	iron sulfide
H_2_S	hydrogen sulfide
Hg	mercury
Hg^0^	elemental mercury
Hg(SR)_2_	mercury-thiol complex
HgS	mercury sulfide
MeHg	methyl mercury
THg	total mercury
XRF	X-ray fluorescence
